# Harnessing 2D and 3D human endometrial cell culture models to investigate SARS-CoV-2 infection in early pregnancy

**DOI:** 10.1042/CS20241215

**Published:** 2025-02-19

**Authors:** Anna Liu, Natalia Ruetalo, Janet P. Raja Xavier, Aditya Kumar Lankapalli, Jakob Admard, Miguel Camarena-Sainz, Sara Y. Brucker, Yogesh Singh, Michael Schindler, Madhuri S. Salker

**Affiliations:** 1 Department of Women’s Health, Eberhard-Karls University Tuebingen, Calwerstr. 7/6, 72076 Tuebingen, Germany.; 2 Institute for Medical Virology, Department of Molecular Virology, University Hospital Tuebingen, Elfriede-Aulhorn-Str. 6, 72076 Tuebingen, Germany.; 3 Ineos Oxford Institute for Antimicrobial Research and Department of Biology, University of Oxford, Oxford OX1 3RE, United Kingdom.; 4 Institute of Medical Genetics and Applied Genomics, Eberhard-Karls University Tuebingen, Calwerstr. 7, 72076 Tuebingen, Germany.

**Keywords:** COVID-19, endometrium, pregnancy, SARS-CoV-2, spheroids

## Abstract

Vertical transmission of SARS-CoV-2 during human pregnancy remains highly controversial as most studies have focused on the third trimester or the peripartum period. Given the lack of early trimester data, determining the prevalence of vertical transmission during early pregnancy and assessing the potential risks for fetal morbidity and mortality pose a challenge. Therefore, we analysed the impact of SARS-CoV-2 infection on an endometrial 3D spheroid model system. The 3D spheroids are capable of decidualization and express angiotensin-converting enzyme 2 (ACE2) as well as transmembrane protease serine 2 (TMPRSS2), rendering them susceptible to SARS-CoV-2 infection. Employing this 3D cell model, we identified that SARS-CoV-2 can infect both non-decidualized and decidualized endometrial spheroids. Infection significantly increased the chemokine Monocyte chemoattractant protein-1 (MCP-1) compared to non-infected spheroids. Decidualized spheroids exhibited upregulated Interleukin (IL)-8 levels. Furthermore, RNA sequencing revealed dysregulation of several genes involved in tissue-specific immune response, Fc receptor signalling, angiotensin-activated signalling and actin function. Gene expression changes varied between SARS-CoV-2 infected non-decidualized and decidualized spheroids and genes associated with the innate immune system (*CD38*, *LCN2* and *NR4A3*) were dysregulated as a potential mechanism for immune evasion of SARS-CoV-2. Altogether, our study demonstrates that endometrial spheroids are a useful model to examine the clinical implications of SARS-CoV-2 vertical transmission, warranting further investigations.

## Introduction

During pregnancy, the female body is subject to profound physiological changes. The immune system is altered to balance immunotolerance of the semiallogenic conceptus whilst providing protection against pathogens [1]. Thus, expectant mothers are more susceptible to some infectious diseases, such as influenza or hepatitis E [2,3]. Pregnant women with COVID-19 have a higher risk of emergency admission, requirement of invasive mechanical ventilation and have more severe cases compared with non-pregnant female patients [4,5].

SARS-CoV-2 can be passed from mother to infant through respiratory droplets during labour or in the postnatal period; however, the question of *in utero* transmission remains unresolved [6]. Infection rates among neonates born to COVID-19-positive mothers are low [7]. Typically, IgM typically does not cross the placental barrier [8]. Nonetheless, reports of early-onset COVID-19 as well as positive IgM antibodies against SARS-CoV-2 in neonates born to women who tested positive for SARS-CoV-2 exist, albeit limited during the early stage of the pandemic [9–11]. Furthermore, SARS-CoV-2 RNA and protein were detected in placentas from mothers with COVID-19 [12,13]. Case reports of miscarriages during the first trimester with positive SARS-CoV-2 RNA and viral proteins found in placental and fetal tissues as well as signs of hyperinflammation indicate the possibility of *in utero* transmission [14,15]. Findings on the impact of SARS-CoV-2 infection on pregnancy outcomes are contradictory with some studies reporting increased risk of miscarriage rates [16,17] and lower in-vitro-fertilisation (IVF) success rates [18] whereas others found no difference compared with non-infected pregnant women [19,20]. While scientific knowledge about COVID-19 is rapidly increasing, many of its effects on early pregnancy remain unknown.

The human endometrium undergoes cyclic transformations to create conducive conditions for embryo implantation [21]. Decidualization occurs in the secretory phase of the menstrual cycle, during which endometrial stromal cells of mesenchymal origin transition into epithelial-like decidual cells [22,23]. Defective decidualization is the cause of a variety of obstetric complications, including miscarriage, preeclampsia and preterm birth [24,25]. The receptor for SARS-CoV-2 cell entry, angiotensin-converting enzyme 2 (ACE2), is expressed in the endometrium and its knockdown results in impaired decidualization [26]. Due to the presence of ACE2 in the human endometrium, SARS-CoV-2 may be able to infect endometrial cells and elicit pathological manifestations, which might increase the risk of early pregnancy loss. However, it is unknown whether SARS-CoV-2 can infect and replicate in human endometrial cells. Importantly, the mere presence of the virus in endometrial cells does not imply endometrial ‘damage’. The primary challenge in answering these key questions lies in the lack of a human disease model of the endometrium capable of replicating infective processes, thus enabling a direct and prospective assessment of viral impact. Here, we established a three-dimensional (3D) spheroid culture of the human endometrium and used two-dimensional (2D) monolayers as reliable models for studying host-pathogen interactions in SARS-CoV-2 infection.

We report that decidualization increases protein levels of SARS-CoV-2 key entry factor ACE2 in the endometrium and that SARS-CoV-2 can infect human endometrial cells and spheroids. We further provide evidence that SARS-CoV-2 infection in these models leads to an inflammatory cytokine response and a dysregulation in genes associated with innate immunity, decidualization and extracellular matrix proteins.

## Materials and methods

### Ethics

Tissue (for the organoids) or SARS-CoV-2 clinical isolate collection was conducted with informed consent, adhering to the Declaration of Helsinki and approved by the Ethics Committee of Eberhard Karls University of Tübingen (Ethical approval 01/2022BO2 and 097/2022A).

### 2D cell culture

Ishikawa cells (ISK), a well-differentiated human endometrial carcinoma cell line (#99040201, Merck, UK) or human endometrial stromal cells (HESC, #T0533, Applied Biological Materials Inc., Canada) were cultured in DMEM/F12 without phenol red medium (Invitrogen, Germany) containing 10% dextran-coated charcoal (DCC, Sigma-Aldrich, USA) stripped fetal bovine serum (FBS, Invitrogen, Germany), 1% antibiotic/antimycotic solution (Invitrogen, Germany) and 1% L-glutamine (Invitrogen, Germany) in a humidified atmosphere at 37°C and 5% CO_2_. Caco-2 (human colorectal adenocarcinoma, ATCC HTB-37) cells were cultured in a humidified atmosphere at 37°C with 5% CO_2_ in DMEM/F12 containing 10% fetal calf serum (FCS) with 2 mM L-glutamine, 100 µg/mL penicillin-streptomycin and 1% non-essential amino acids (Invitrogen, Germany).

### 3D spheroid culture

HESC and ISK cells were cultured in cell culture flasks until reaching 80% confluency. Two-dimensional (2D) cells were detached with trypsin-EDTA (Invitrogen, Germany), then suspended in medium, centrifuged and counted. Cells were seeded onto 96-well flat bottom plates coated with 1% (w/v) agarose (Invitrogen, Germany) at a density of 6000 HESC and 3000 ISK cells per 100 μl per well. The spheroid culture was maintained in DMEM/F12 without phenol red medium supplemented with 5% DCC FBS, 1% antibiotic/antimycotic solution and 1% L-glutamine in a humidified atmosphere at 37°C and 5% CO_2_. The medium was replenished every two to three days.

### Endometrial organoid culture

Organoids were derived from primary stem cells obtained from tissue biopsies of healthy premenopausal subjects at the University Women’s Hospital Tübingen. Tissue samples were processed and organoids were cultured according to the previously described protocol [27]. Images were captured over nine days using the EVOS M7000 (ThermoFischer, Germany) with tile-stitching.

### Decidualization treatment

HESC and ISK cells were cultured until reaching 80% confluency, then seeded into six-well plates at a concentration of 1 × 10^5^ cells/mL with 1 mL per well and cultured for two days. Spheroids were cultured for two days in a 96-well plate. Organoids were cultured in 48-well plates to 80% confluency. Subsequently, the cells were treated with 1 μM medroxyprogesterone 17-acetate (MPA, #M1629, Sigma-Aldrich, Germany) and 0.5 mM 8-bromo-cAMP (#1140, Tocris, UK) in 2% DCC FBS DMEM/F12 medium for monolayer cells and in 5% DCC FBS DMEM/F12 medium for spheroids. Treatment was performed every 48 h for a total of six days before cells were used for downstream experiments.

### *In vitro* viral infection

All experiments involving authentic SARS-CoV-2 viruses were conducted in a Biosafety Level 3 laboratory. For the determination of multiplicity of infection (MOI) a titration using serial dilutions of viral stocks was conducted.

ISK cells were cultured on six-well plates with 1 × 10^5^ cells per 1 mL per well. Prior to infection, half of the cells were treated with decidualization medium for six days. Cells were infected with recombinant SARS-CoV-2 expressing mNeonGreen (SARS-CoV-2-mNG [28,29]) at an MOI of 10 or with wildtype B.1 SARS-CoV-2 variant (isolated from a throat swab sample at the Institute for Medical Virology and Epidemiology of Viral Diseases, University Hospital Tübingen [29], referred to as B1 in the text and figures) at an MOI of 40. Control wells were mock-infected. Infection was monitored by fluorescence microscopy and pictures were taken 48 and 72 hours post infection (hpi). Cells were then either fixed for immunofluorescence microscopy (IF) or harvested for Western blot (WB).

Endometrial spheroids were cultured in agarose-coated 96-well plates for seven days and treated with decidualization medium for five days prior to infection. Spheroids were infected with SARS-CoV-2 B.1.617.2 (isolated from a throat swab collected in May 2021 at the Institute for Medical Virology and Epidemiology of Viral Diseases, University Hospital Tübingen [30]) at a concentration of 648,267,898 IU/mL (diluted 1:20). Supernatant was collected and remaining viral particles were inactivated with UV-C light (254 nm) for 10 min using the Soluva pro UV Disinfection Chamber (Heraeus, Hanau, Germany). Spheroids were collected, washed with PBS and lysed using Radio-Immunoprecipitation Assay (RIPA) Lysis Buffer (for protein extraction, see below), lysis buffer (for RNA extraction) or fixed in 4% paraformaldehyde (PFA, for immunostaining). After inactivation, samples were further processed in an S2 biosafety laboratory.

### Western blotting

Whole-cell protein extracts were obtained by lysis using RIPA buffer (10 mM Tris-HCl pH 7.4, 150 mM NaCl, 1% Triton X-100, 0.1% Na-deoxycholate, 0.1% SDS, 1 mM EDTA, 0.5 mM EGTA, Protease Inhibitor Cocktail Tablets, EDTA-Free - all purchased from Sigma-Aldrich, Germany). After protein extraction samples, were mixed with Laemmli buffer and boiled at 95°C for 5 min. Proteins were separated by electrophoresis on hand-casted 10% SDS-polyacrylamide gels and transferred to polyvinylidene difluoride (PVDF) membranes (Sigma-Aldrich, Germany). Non-specific binding sites were blocked with 5% non-fat dry milk in Tris-buffered saline with 0.1% Tween™20 (TBST) for 1 h at room temperature. Membranes were incubated overnight at 4°C with primary antibodies against ACE2 (#21115–1-AP, Proteintech, Germany; 1:700), transmembrane protease serine 2 (TMPRSS2) (#14437–1-AP, Proteintech, Germany; 1:1000), SARS-CoV-2 nucleocapsid (#40143-R001, SinoBiological, Germany; 1:1000) and anti-Glyceraldehyde-3-phosphate dehydrogenase (GAPDH, #2118, Cell Signaling Technology, Germany; 1:2000). Afterwards, membranes were washed three times with TBST, followed by incubation with Horseradish peroxidase (HRP)-conjugated anti-rabbit secondary antibodies (#7074, Cell Signaling Technology, Germany; 12000). Protein complexes were visualized by chemiluminescence using WesternBright enhanced luminol-based chemiluminescent (ECL) HRP substrate (Advansta, Germany) and bands were quantified using the Image J (v.1.53k) software. Relative protein quantity was compared with the non-decidualized and/or non-infected control group, for which the quantity was standardized to 1.0.

### RNA isolation, cDNA synthesis and qPCR

Total RNA was extracted from cells and spheroids using RNeasy Mini Kit (#74104, Qiagen, Germany) according to the manufacturer’s protocol. cDNA synthesis was performed with Maxima H Minus cDNA Synthesis Master Mix (#M1681, Thermofisher Scientific, Germany), followed by quantitative (qPCR) using SYBR Green (#A25742, Thermofisher Scientific, Germany) with the QuantStudio 3 PCR system (Thermofisher Scientific, Germany). Primers shown in Table 1 were used to detect *PRL* [31]*, IGFBP1* [32]*, HSD11B1* [33]*, CCL20* [34]*, CD38* [35]*, LCN2* [36]*, VTCN1* [37]*, NR4A3* [38], *LEFTY1* [39] *and ACTB* [40]. Relative gene expression levels were determined using the 2^−ΔΔCt^ method, with normalization to the reference gene *ACTB* (*beta-actin*) and the non-decidualized and/or non-infected control sample. The expression level of the control group was defined as 1.0. All measurements were performed in triplicates.

**Table 1:** Human primers used for qPCR.

Gene	Forward primer	Reverse primer
*ACTB*	5’-ATGGAGAAAATCTGGCACCAC-3’	5’-TTGAAGGTCTCAAACATGATCTGG-3’
*CCL20*	5’-TGTGCGTCTCCTCAGTAAAAA-3’	5’-ACAAGTCCAGTGAGGCACAA-3’
*CD38*	5’-AGCACTTTTGGGAGTGTGGAA-3’	5’-GATCCTGGCATAAGTCTCTGGA-3’
*HSD11B1*	5′-AGCAAGTTTGCTTTGGATGG-3′	5′-AGAGCTCCCCCTTTGATGAT-3′
*IGFBP1*	5’-CGAAGGCTCTCCATGTCACCA-3’	5’-TGTCTCCTGTGCCTTGGCTAAAC-3’
*LCN2*	5’-CACCTCCGTCCTGTTTAGGAAA-3’	5’-TGCTGGTTGTAGTTGGTGCT-3’
*LEFTY1*	5’-TGGACAAATGCTCTGTGCTCT-3’	5’-TCCAGTGGCCAAAGATTCTCA-3’
*NR4A3*	5’-GCAAGATACCCTCCAGATATGC-3’	5’-TTGGTGTAGTCGGGGTTCAT-3’
*PRL*	5’-AAGCTGTAGAGATTGAGGAGCAAAC-3’	5’-TCAGGATGAACCTGGCTGACTA-3’
*VTCN1*	5’-GCAGATCCTCTTCTGGAGCATAA-3’	5’-AGTGCAGCTCAGGATTCCAT-3’

### Immunofluorescent staining (IF) of ISK and HESC cells after infection

Cells infected with B.1 SARS-CoV-2 in six-well plates were fixed with 80% acetone for 5 min, washed with PBS and blocked for 1 h at room temperature (RT) with 10% normal goat serum (NGS). IF staining was performed using an anti-SARS-CoV-2 nucleocapsid antibody (GTX135357, GeneTex/Biozol, Germany; 1:1000) and goat anti-rabbit Alexa594-conjugated secondary antibody. Alternatively, cells infected with SARS-CoV-2-mNG were fixed with 2% PFA for 10 minutes at 37°C. For quantification of infection rates, images were taken with the Cytation3 (BioTek) and Alexa 594-positive cells as well as the total number of cells (bright field) were automatically counted by the Gen5 software v.3.10 (BioTek).

### Immunofluorescent staining of endometrial spheroids

The protocol of Weiswald et al. [41] was followed with slight modifications. All steps were carried out at 4°C. Endometrial spheroids were collected, centrifuged, washed with PBS (Sigma-Aldrich, Germany) and fixed with PBS containing 4% PFA (Thermofisher Scientific, Germany) and 1% Triton X-100 (Thermofisher Scientific, Germany) for 3 h. Spheroids were washed three times with PBS and dehydrated in ascending concentrations of methanol (Honeywell, USA) in PBS (25%, 50%, 75% and 95%) for 30 min each at 4°C, followed by 5 h in 100% methanol. Rehydration was carried out with descending concentrations of methanol in PBS (95%, 75%, 50% and 25%) for 30 min each. Spheroids were washed three times in PBS and blocked with phosphate-buffered saline with 0.1% Triton X-100 (PBST) containing 3% BSA (Thermofisher Scientific, Germany) overnight. After washing with PBST, spheroids were incubated with primary antibodies against cytokeratin 7 (#4465, rabbit anti-human, Invitrogen, Germany; 1:100) and vimentin (#60330–1-Ig, mouse anti-human, Proteintech, Germany; 1:50) or SARS-CoV-2 nucleocapsid (#40143-R001, SinoBiological, Germany; 1:100), respectively, for 48 h on a rotator. Spheroids were washed four times with PBST, incubated with secondary antibodies (Alexa Fluor 488 anti-rabbit (#A-11008, 1:300) and Alexa Fluor 594 anti-mouse (#A-11005, 1:400) or Alexa Fluor 594 anti-rabbit (#A-11012, 1:400), all from Invitrogen, Germany, respectively) for 24 h and washed three times in PBST. For infected spheroids, sequential staining was performed using primary antibodies against ACE2 (#21115–1-AP, Proteintech, Germany; 1:100) for 48 h on a rotator, followed by four wash steps and incubation with secondary antibodies (Goat anti-Rabbit IgG Alexa Fluor 488, #A-11008, Invitrogen, Germany; 1:400). After washing and mounting using 90% glycerol (v/v), slides were imaged using the EVOS M7000 microscope (ThermoFisher, Germany).

### RNA extraction, library preparation, sequencing and data analysis

The PureLink RNA Mini Kit (#12183020, Invitrogen, Germany) was used for RNA extraction and the manufacturer’s manual was followed. RNA sequencing (RNA-seq) libraries were generated using the NEBNext Ultra II Directional RNA Library Prep Kit for Illumina, with 100 ng of RNA utilized for each library. The library preparation protocol followed the manufacturer’s instructions and involved poly(A) selection to enrich for mRNA transcripts. The Illumina NovaSeq 6000 platform was employed for sequencing in paired-end mode with read length of 50 bp and an approximate depth of 70 million clusters per library. To minimize technical batch effects, library preparation and sequencing procedures were performed by the same individual. The quality of raw RNA-seq data in FASTQ files was assessed using ReadQC (ngs-bits version 2018_06) to identify potential sequencing cycles with low average quality and base distribution bias. Subsequently, reads were preprocessed using skewer (v.0.2.2) and aligned to the human reference genome (GRCh37) with STAR (v.2.5.4a), allowing spliced read alignment. Alignment quality was further evaluated using MappingQC (ngs-bits v. 2018_06) and visually inspected using the Broad Integrative Genome Viewer (v.2.3.1). For gene-level quantification, read counts were obtained using Subread (v.1.6.0) and the Ensembl genome annotation (GRCh37 v.75).

For the differential gene expression analysis, raw gene read counts were filtered to retain genes with at least 1 count per million in at least three samples. This filtering step resulted in more than 19,000 genes being considered for determining differential expression in the pair-wise comparisons between experimental groups. The analysis was conducted using edgeR (v.3.22.3), which employs a statistical framework based on negative binomial distributions and gene-wise testing through generalized linear models. We used log2 fold change >1.5 and raw P-value <0.05 for the Volcano plots using the EnhancedVolcano package (R studio) and Heatmap for the selected genes. Data are available under Gene Expression Omnibus (GEO) accession ID (GSE274209).

### Gene set enrichment analysis

Gene set enrichment analysis (GSEA) was performed in R using fgsea [42] and DOSE packages [43].

### *In silico* analysis

The data used for the analyses described in this manuscript were obtained from the GTEx Portal on 05/23/23 dbGaP accession number phs000424.vN.pN.Re-analysis of single cell-RNA-seq (GSE111976) using Seurat package in R (v.5.1.0) was performed based on the data described [44]. Data were downloaded from https://www.ebi.ac.uk/biostudies/arrayexpress/studies/E-MTAB-6701 with experiment codes, E-MTAB-6701 (for droplet-based data) and E-MTAB-6678 (for Smart-seq2 data).

### Cytokine quantification through flow cytometry

Supernatant samples were exposed to UV-C light (254 nm) in a UV disinfection chamber (Heraeus, Germany) for 10 min to inactivate viral particles. The LEGENDplex Human Inflammation Panel 1 (13-plex) with V-bottom Plate (#740809, BioLegend, USA), a multiplex bead-based assay, was used to prepare the samples for cytokine quantification through flow cytometry according to the manufacturer’s protocol. The samples were acquired on a flow cytometer (BD Fortessa) and the data were analysed using the Data Analysis Software Suite for LEGENDplex™ (https://www.biolegend.com/de-de/immunoassays/legendplex/support/software).

### Statistical analysis

Images were prepared in GraphPad (v.7), R and/or Inkscape (v.1.4). Statistical analysis was performed using GraphPad Prism software. Normality was assessed with a Shapiro-Wilk test and a QQ-plot. Cytokine levels in SARS-CoV-2-infected endometrial spheroids were analysed via analysis of variance (ANOVA) followed by Tukey’s test. RNA-seq results were evaluated with the Quasi-likelihood F-test followed by the Benjamini-Hochberg method for multiple testing correction. Genes with adjusted *P*-value (false discovery rate (FDR)) < 0.05 and fold change ≥1.5 were considered significantly differentially expressed. Statistical analysis was performed using the unpaired two-tailed student’s t-test except for the qPCR validation which was performed with multiple comparisons using the Kruskal-Wallis test. Data are presented as mean ± SEM. Significance levels: *P < 0.05, **P < 0.01, ***P < 0.001, ****P < 0.0001.

## Results

### SARS-CoV-2 entry factors ACE2 and TMPRSS2 are present in the endometrium

To determine the presence of SARS-CoV-2 entry factors ACE2 and TMPRSS2 in the female reproductive tract, which is the basic requirement for a potential permissiveness of this tissue for SARS-CoV-2 infection, we performed *in silico* analysis of transcripts on the publicly available database GTEx portal. Our findings show a similar expression of ACE2 transcripts in both the uterus and vagina compared with the lung (Supplementary Figure S1). Conversely, TMPRSS2 transcript levels were notably abundant in the vagina and less so in uterine tissue. Further, we investigated expression levels of ACE2 and TMPRSS2 in endometrial tissue using publicly available single-cell data from the endometrium using 71,032 cells (GSE111976). The presence of ACE2 was observed in ciliated (1.27%), epithelial (< 1.5%) and stromal cells (< 0.5%), while TMPRSS2 was highly expressed in epithelial (~10%) and ciliated cells (~10%) (Supplementary Figure S2). Co-expression of ACE2 with TMPRSS2 was observed only in epithelial cells but was at low abundance (0.07%). Based on these observations, we hypothesized that the female reproductive tract may be susceptible to SARS-CoV-2 infection.

### Expression of the SARS-CoV-2 entry factors ACE2 and TMPRSS2 in human endometrial cells

To assess if the human endometrium is a potential target of SARS-CoV-2 replication, we made use of an *in vitro* infection model. To this end, we utilized ISK, which was previously shown to recapitulate the phenotype and function of endometrial epithelial cells [45], as well as HESC [46]. To determine whether ISK and HESC could serve as a model to study the host-pathogen interaction of the human endometrium with SARS-CoV-2, we first evaluated by Western blot whether these cell types express the surface markers ACE2 and TMPRSS2, known to serve as key viral entry factors for SARS-CoV-2. The colorectal adenocarcinoma cell line Caco-2, frequently used as a coronavirus cell culture model with known ACE2 and TMPRSS2 expression [47] served as a positive control.

**Figure 1:**
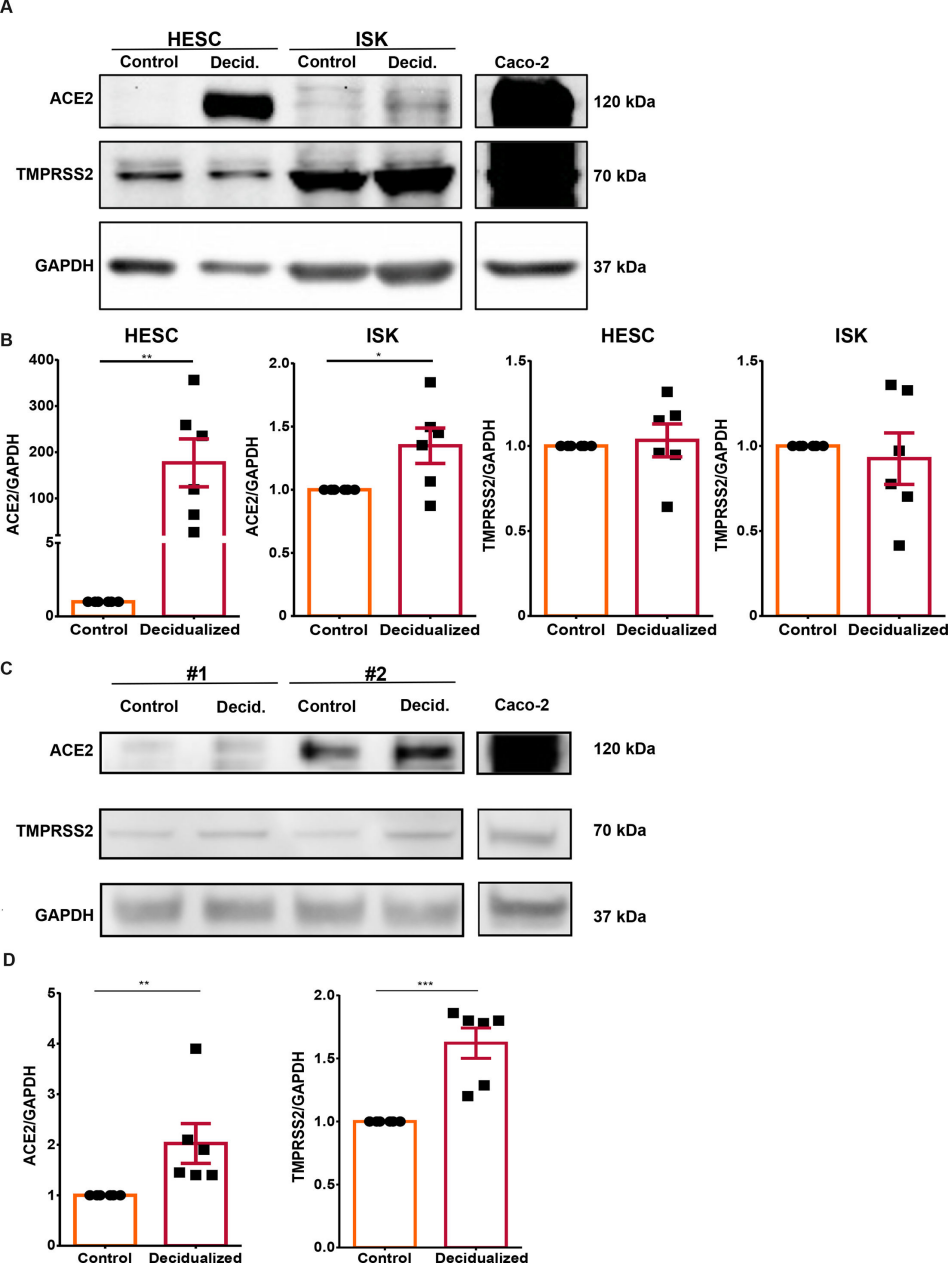
**ACE2 and TMPRSS2 protein levels in endometrial 2D cells (monolayers) and 3D spheroids**. HESC and ISK (*n* = 6) were cultured as monolayers or as spheroids and treated with decidualizing stimuli medroxyprogesterone acetate (1 µM; MPA) and 8-Bromo-cAMP (0.5 mM) every 48 h for six days. The cells were lysed and protein was extracted. Western blotting was performed for the 2D cultures (A, B) and two independent spheroid cultures (C, D) with GAPDH as loading control and Caco-2 as positive control. Relative expression of ACE2 and TMPRSS2 was calculated and unpaired student’s t-tests were performed. Data are presented as arithmetic means ± SEM. *P < 0.05; **P < 0.01. Each point represents an independent biological experiment performed.

In non-decidualized HESC or ISK, ACE2 protein levels were either below the detection limit or low, respectively (Figure 1A). We further investigated whether treatment with MPA and 8-Bromo-cAMP, known to induce decidualization [48], influences levels of ACE2 and TMPRSS2 in ISK and HESC. Interestingly, ACE2 was significantly increased 177.0-fold (*P*<0.01) in HESC and 1.3-fold (*P*<0.05) in ISK after decidualization treatment (Figure 1B). In both cell types, TMPRSS2 was present and levels did not change upon decidualization (Figure 1B). A significant increase in RNA expression of decidualization markers prolactin, IGFBP-1 and 11β-HSD1 in HESC confirmed successful decidualization (Supplementary Figure S3A), whereas these markers were either downregulated or remained unchanged in ISK (Supplementary Figure S3B).

Considering that the key entry factors for SARS-CoV-2 are expressed in HESC and ISK, albeit at low and differential levels, we next used these two cell types and the liquid-overlay technique to establish a novel three-dimensional spheroid model of the human endometrium. In contrast to cells grown in a monolayer, 3D cell culture allows for complex cell-cell interactions resulting in improved representation of the *in vivo* tissue-specific phenotype and organ function [49,50]. Successful formation and growth of endometrial spheroids were monitored through phase-contrast microscopy (Supplementary Figure S4A) and immunostaining confirmed the presence of both cytokeratin 7, a known epithelial cell marker [51,52], and vimentin, a known marker for stromal cells [53,54] (Supplementary Figure S4B). ACE2 and TMPRSS2 were shown to be present in the endometrial spheroids through Western blotting (Figure 1C, two independent spheroids are presented) and their levels increased with decidualization (Figure 1D). Furthermore, decidualization markers prolactin, IGFBP-1 and 11β-HSD1 were monitored in spheroids and confirmed successful decidualization (Supplementary Figure S4C).

### SARS-CoV-2 infects endometrial monolayers and spheroids

**Figure 2:**
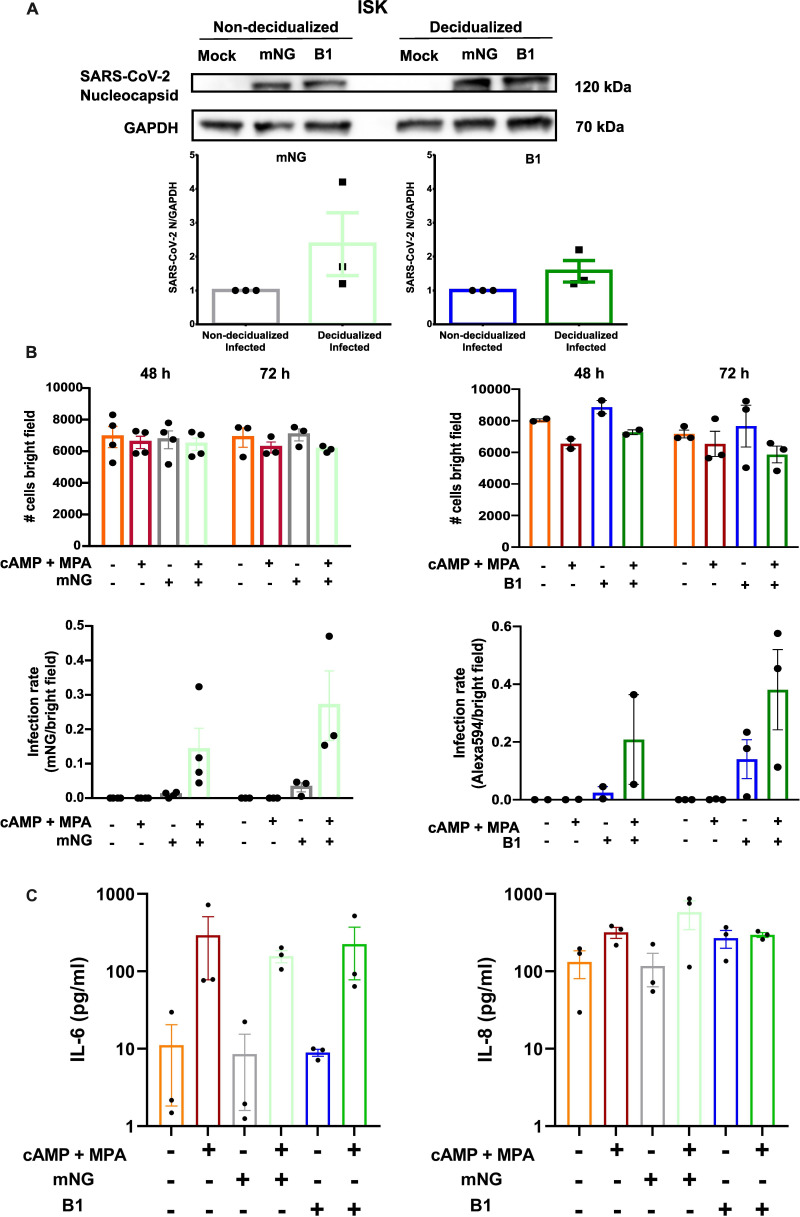
**Infection of 2D Ishikawa cells with SARS-CoV-2**. (**A**) ISK cells were treated with or without cAMP and MPA and infected with SARS-CoV-2-mNG or the clinical isolate B1. After infection, cells were collected for protein extraction and western blotting. Detection of SARS-CoV-2 proteins was performed using an anti-nucleocapsid antibody. GAPDH was used as house-keeping protein. Western blot quantification can be seen in the lower panel (*n* = 3). (**B**) Upper panels showed the total cell count (bright field) for the corresponding experiments. Lower panels show the infection rates calculated as infected cells/total number of cells (*n* = 2–4). (**C**) Supernatant was collected 72 hpi and cytokines were assayed (*n* = 3) for IL-6 and IL-8 presented in log10 scale.

To assess the potential for vertical transmission of SARS-CoV-2 during pregnancy, we studied the infectivity of SARS-CoV-2 in both endometrial 2D cell cultures and 3D spheroids. ISK monolayers were cultured either in control or decidualization medium and were either infected with SARS-CoV-2-mNG, a SARS-CoV-2 reporter virus exhibiting green fluorescence upon productive infection, or with the B.1 strain of SARS-CoV-2 from a clinical isolate. Infection of 2D ISK cells was confirmed for both viruses by the presence of the SARS-CoV-2 nucleocapsid protein in Western blot assays (Figure 2A). In addition, fluorescence microscopy was used to follow infection with the reporter virus, while IF was performed to evaluate infection with the clinical isolate (B1). mNG-expressing cells or SARS-CoV-2 nucleocapsid fluorescence are shown in Supplementary Figure S5. Infection rates were calculated at 48 and 72 hpi (Figure 2B and Supplementary Figure S5). Decidualization tended to increase SARS-CoV-2 nucleocapsid levels inside infected cells, as shown by Western blotting (Figure 2A). This was further confirmed by the calculation of infection rates which were increased after decidualization for both viruses at both time points (Figure 2B and Supplementary Figure S5). We next assessed if SARS-CoV-2 viral infection can lead to a cytokine-storm-like phenotype during the decidualization process. We observed that decidualization increases IL-6 and IL-8 levels, while infection with SARS-CoV-2 did not further augment these cytokines (Figure 2C).

**Figure 3:**
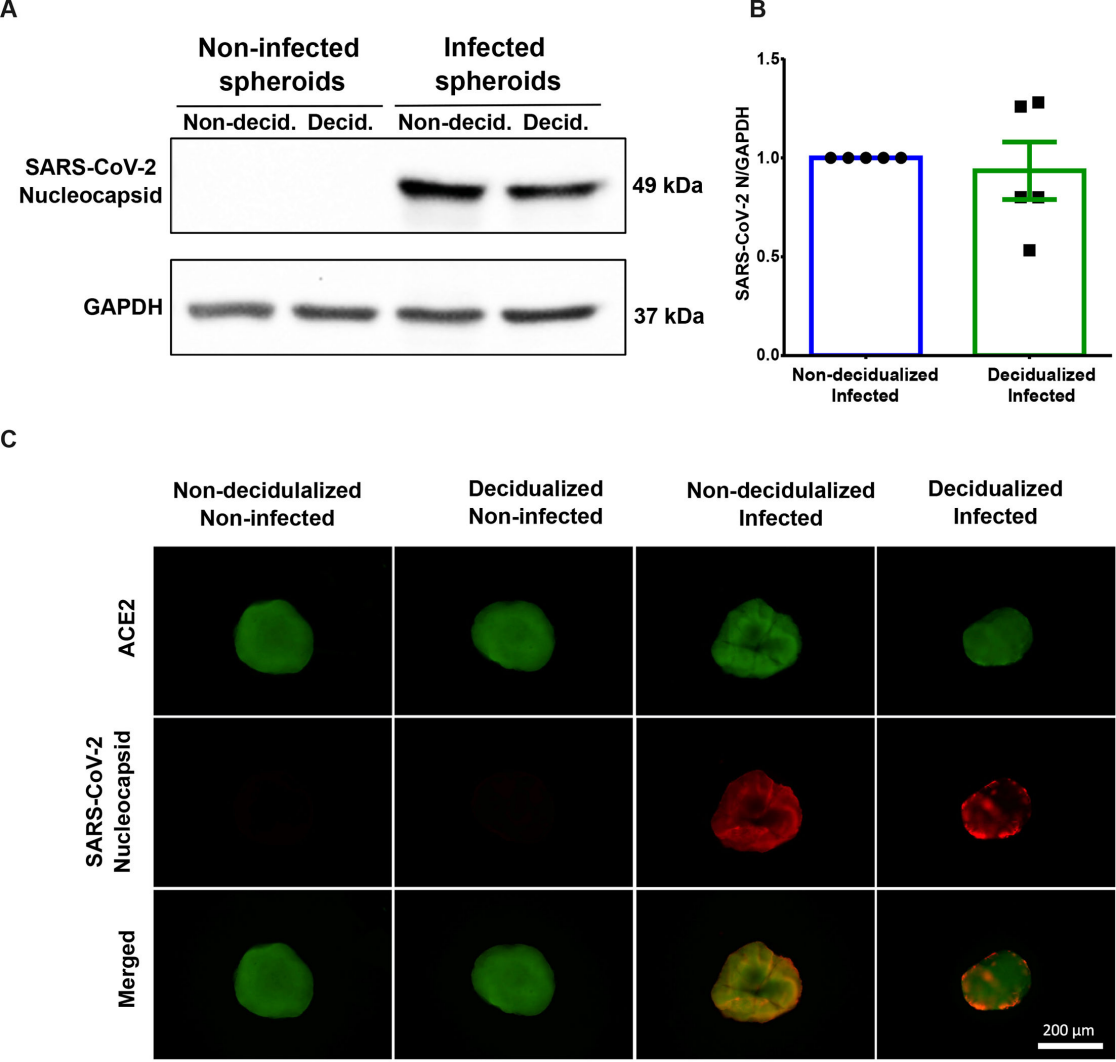
**Infection of endometrial spheroids with SARS-CoV-2**. HESC and ISK were cultured separately before being added to agarose-coated wells of a 96-well plate for spheroid formation. After two days, the spheroids were treated with 8-Bromo-cAMP and MPA, which was repeated every 48 h. On day seven, spheroids were infected with SARS-CoV-2 B.1.617.2 and spheroids were collected 48 hpi for downstream analysis. Western blotting (**A, B**) was performed to detect SARS-CoV-2 nucleocapsid protein (*n* = 5). GAPDH was used as an internal loading control. SARS-CoV-2 nucleocapsid protein levels were compared between infected non-decidualized and decidualized samples. Immunostaining of endometrial spheroids was performed for ACE2 and SARS-CoV-2 nucleocapsid protein (**C**; images are representative of three independent experiments). Scale bar is 200 µM.

Given that monolayers of ISK cells are permissive to infection, we next analysed the infectivity of 3D spheroid cultures. Endometrial spheroids were cultured for seven days and then decidualized for six days prior to infection with SARS-CoV-2 B.1.617.2, a clinical isolate of the delta variant. To verify infection, SARS-CoV-2 nucleocapsid protein was detected through Western blotting (Figure 3A and B) and immunofluorescence (Figure 3C). As seen in Figure 3C, immunoreactivity against ACE2 was observed in all four experimental groups. A nucleocapsid signal was observed for the infected spheroids compared to the non-infected ones and no significant difference was observed between infected non-decidualized and decidualized spheroids (Figure 3B and C). We futher tested the use of endometrial-derived organoids, however, the results showed that ACE2, the functional receptor necessary for SARS-CoV-2 infection, is not present (Supplementary Figure S6). In sum, we conclude that endometrial monolayers and spheroids can be infected in normal and decidualized conditions.

### Infection with SARS-CoV-2 results in endometrial inflammation

**Figure 4:**
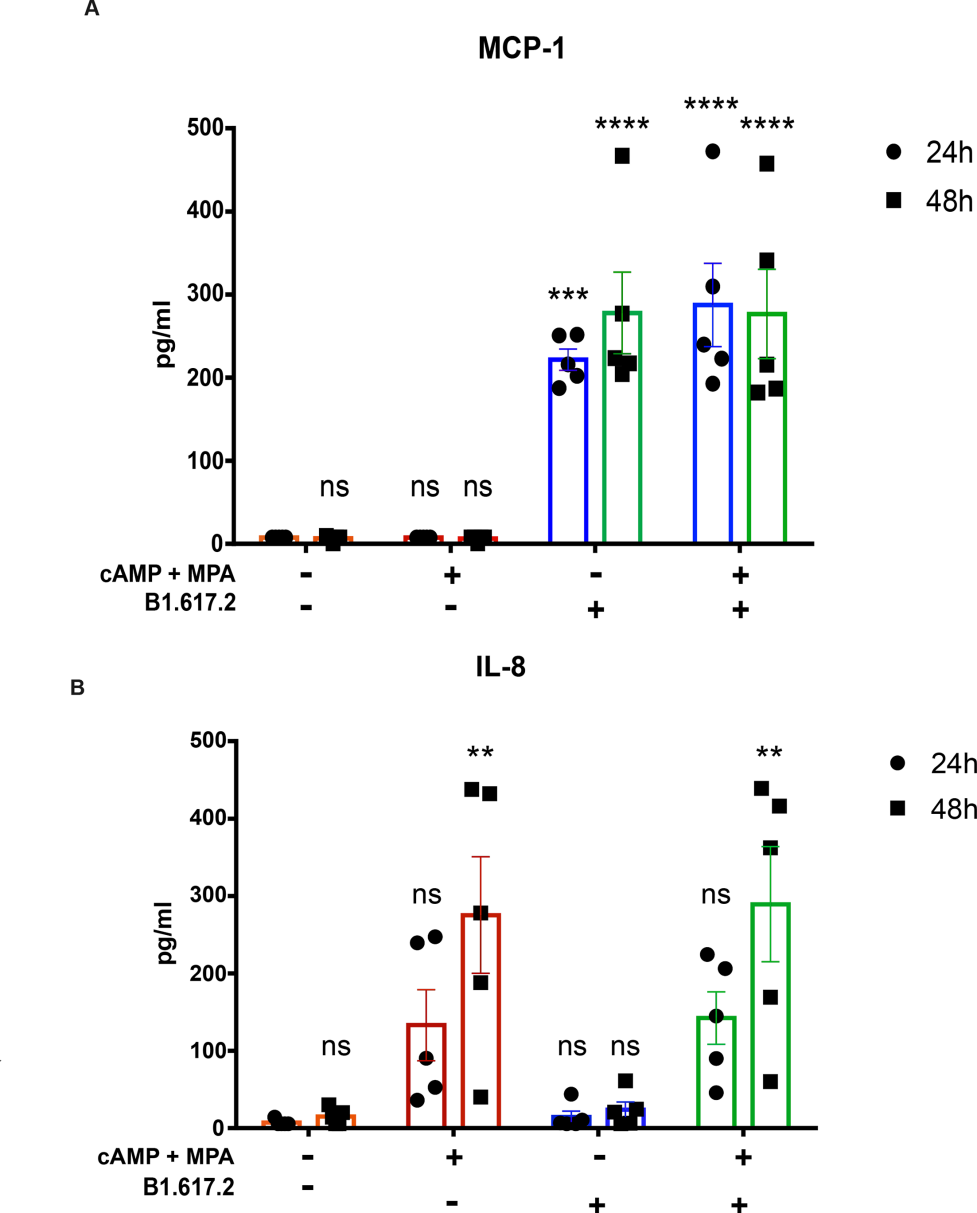
**Cytokine levels of infected and non-Infected endometrial spheroids**. Endometrial spheroids (*n* = 5) were cultured for seven days and decidualized for five days prior to infection with SARS-CoV-2 B.1.617.2. Supernatant was collected 24 hpi and 48 hpi and cytokines MCP-1 and IL-8 were measured through flow cytometry (A, B). ANOVA was used for statistical analysis. Data are presented as arithmetic mean ± SEM. Degree of statistical significance is shown in comparison to non-decidualized non-infected endometrial spheroids. **P < 0.01; ***P < 0.001; ****P < 0.0001; ns, non-significant.

To examine the inflammatory response elicited by SARS-CoV-2 infection in the endometrial spheroids, cytokine levels were measured in the supernatant 24 and 48 hpi using a multiplex bead-based assay coupled with flow cytometry (Figure 4A and B). Of the 13 assayed cytokines, only two were above the detection level. We observed that MCP-1, a proinflammatory chemotactic chemokine, was significantly increased in infected compared to non-infected samples (*P*<0.001), whereas decidualization had no significant impact on the level of MCP-1. In contrast, chemokine IL-8, another inflammatory mediator, was not significantly changed after infection but we observed a significant increase in the decidualized spheroids after 48 hpi compared to the non-decidualized spheroids (*P*<0.01).

### SARS-CoV-2 infection causes dysregulation of genes associated with immune response in endometrial spheroids

**Figure 5:**
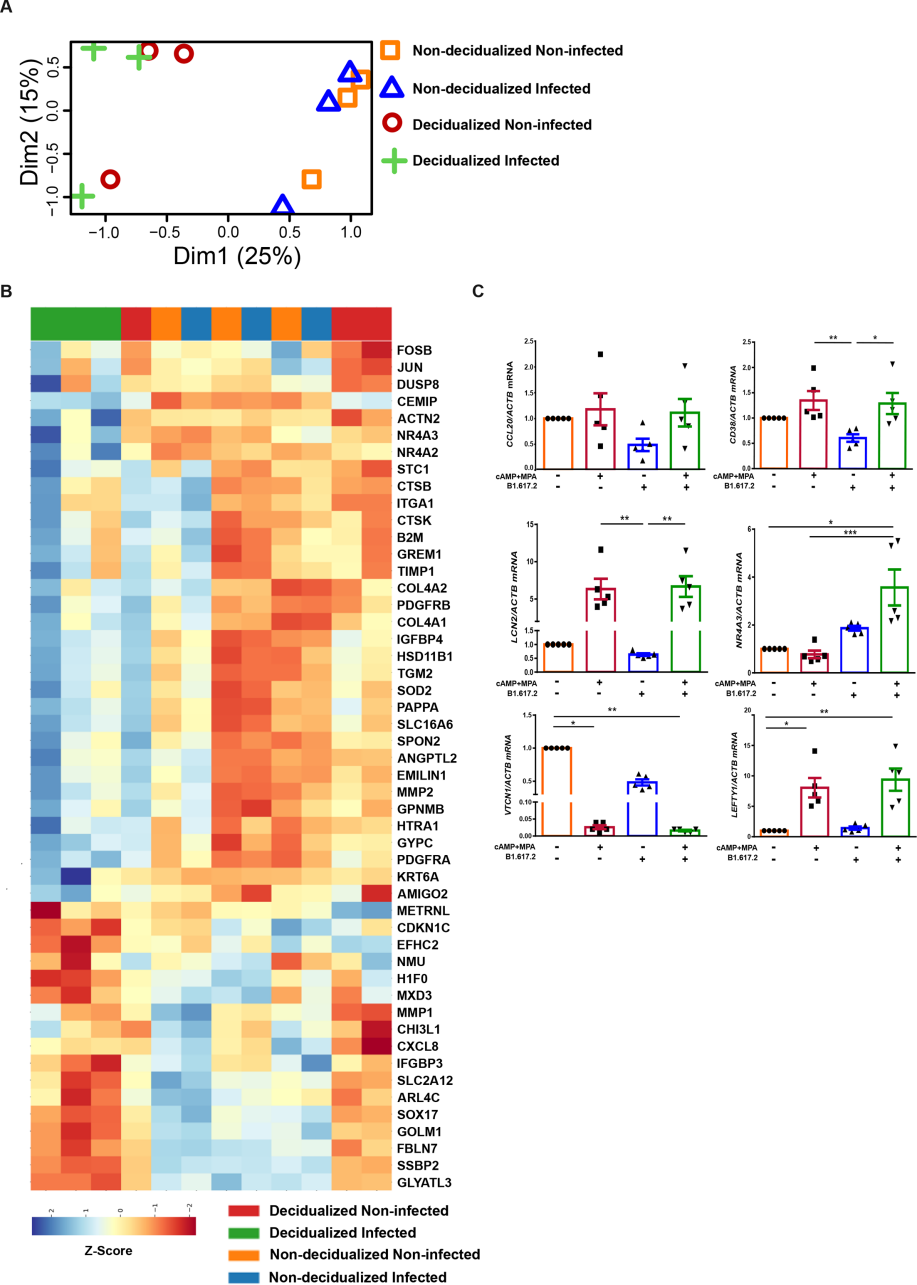
**DEGs in endometrial spheroids with and without SARS-CoV-2 infection**. Endometrial spheroids (*n* = 3) were treated with 8-Bromo-cAMP and MPA for five days prior to infection with SARS-CoV-2 B.1.617.2. RNA was extracted 48 hpi and RNA sequencing was performed. (**A**) Dimensional reduction analysis shows variance in mRNA expression between the experimental groups. (**B**) Top 50 DEGs are presented in a heatmap. An independent set of decidualized and non-decidualized endometrial spheroids (*n* = 5) were infected with SARS-CoV-2 B.1.617.2 and RNA was extracted after 48 h of incubation. (**C**) qPCR was performed for *CCL20*, *CD38*, *LCN2, NR4A3*, *VTCN1* and *LEFTY1* with *ACTB* as reference gene. Statistical analysis was performed with multiple comparisons using the Kruskal-Wallis test. Data are presented as arithmetic mean ± SEM. *P < 0.05; **P < 0.01; ***P < 0.001.

To assess the effect of SARS-CoV-2 infection on gene expression, we conducted RNA sequencing to identify gene expression profiles in non-infected and infected as well as non-decidualized and decidualized endometrial spheroids. Firstly, we analysed the variance in mRNA expression between the experimental groups (non-decidualized non-infected, non-decidualized infected, decidualized non-infected and decidualized infected) using principal component analysis. The cumulative variance plot demonstrated a distinction between non-decidualized and decidualized groups, whilst the separation based on infection status is less pronounced in non-decidualized samples, it becomes more distinct in decidualized spheroids (Figure 5A). A heatmap presents the top 50 differentially regulated genes in the different experimental groups (Figure 5B and Supplementary Table S1) and volcano plots show group comparisons (Supplementary Figure S7A-D). Focusing on selected differentially expressed genes (DEGs), another heat map (Supplementary Figure S7E) displays the expression patterns in pairwise comparisons of the experimental groups. Comparisons between each group provide insights into the specific gene expression changes associated with decidualization treatment (first and second column) and infection (third and fourth column). Secondly, we measured the effect of infection based on differential gene expression. As a result, we identified 84 unique genes in non-decidualized (infected vs non-infected) samples and 132 unique genes in decidualized (infected vs non-infected) samples. There were 23 overlapping genes between these experimental groups (Supplementary Figure S7F).

Based on the RNA-seq analysis, we observed that in non-decidualized spheroids, infection led to an increased fold change expression of *TNFSF18* and *LEFTY1* (Supplementary Figure S7A). Futhermore, in infected decidualized samples we observe a higher fold change *NR4A3* and lower complement *C7* expression (Supplementary Figure S7B). The effect of decidualization can be seen by an upregulation of *IL1RL1* and *PECAM1* in both non-infected and infected samples (Supplementary Figure S7C,D). Notably, *VTCN1* was downregulated in decidualized non-infected spheroids and futher reduced in decidualized infected samples (Supplementary Figure S7C). Taken together, these results show that both infection and decidualization impacted the gene expression profiles of the endometrial spheroids (Supplementary Figure S7). To validate the RNA-seq results in an independent experiment, qPCR was performed on a subset of selected genes that were regulated in decidualized infected spheroids (Figure 5C). Endometrial spheroids (*n* = 5) were cultured and decidualized and/or infected as in previous experiments or remained untreated. Three genes, *CCL20* (0.48-fold)*, CD38* (0.61-fold) and *LCN2* (0.63-fold) were downregulated in non-decidualized spheroids upon SARS-CoV-2 infection (non-decidualized non-infected vs non-decidualized infected) although not reaching statistical significance. For *CD38* and *LCN2*, gene expression was also significantly higher in the decidualized infected compared with the non-decidualized infected samples (2.30-fold, *P*=0.0226 and 13.35-fold, *P*=0.0042, respectively). *NR4A3* expression increased in infected samples, particularly in decidualized spheroids (3.57-fold, *P*=0.001) compared with non-infected decidualized spheriods. *LEFTY1* expression was significantly higher in decidualized infected spheroids when compared with non-decidualized non-infected spheriods (9.3-fold, *P*=0.0090). *VTCN1* was significantly downregulated by both infection and decidualization. Overall, the qPCR analysis from independent samples verified the RNA-sequencing results.

**Figure 6:**
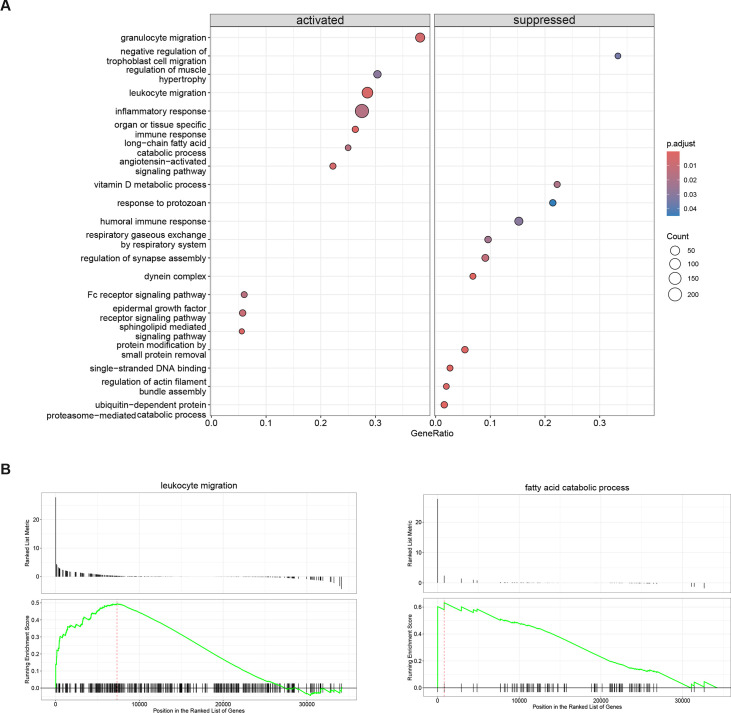
Gene set enrichment analysis. (**A**) GSEA against the KEGG database for differentially enriched pathways in genes in endometrial spheroids with and without SARS-CoV-2 infection. Enrichment plots for the two pathways upregulated in the experiments are shown. (**B**) The relative gene positions are indicated by the straight lines (line plot) under each graph. Lines clustered to the left of the red dotted line represent higher-ranked genes in the ranked list and contribute to core enrichment for each pathway.

Further, GSEA allows for an interpretation of gene expression through predefined gene set databases and is a critical step in understanding different phenotypes. Decidualized spheroids simulate the endometrium during early pregnancy, thus, we focussed only on decidualized infected and decidualized non-infected samples. We identified that several pathways were upregulated only in decidualized infected spheroid samples, such as granulocyte migration, regulation of muscle hypertrophy, leukocyte migration, organ or tissue-specific immune response, long-chain fatty acid catabolic process, angiotensin-activated signalling pathway, Fc receptor signalling pathway, epidermal growth factor receptor signalling pathway and sphingolipid mediated signalling pathway (Figure 6A). Interestingly, vitamin D metabolic process, humoral immune response, respiratory gaseous exchange, regulation of synapse assembly and actin filament bundle assembly as well as negative regulation of trophoblast cell migration were suppressed. Moreover, we present the leukocyte migration pathway which has an enrichment score of 0.49, normalized enrichment score (NES) of 1.45 and a *P*-value of 0.003 as well as the long-chain fatty acid catabolic process with an enrichment score of 0.90, NES of 1.55 and a *P*-value of 0.01 (Figure 6B). Overall, our GSEA analysis suggested that SARS-CoV-2 infection during decidualization increases inflammatory responses and simultaneously suppresses the humoral immune response which could help the virus to evade the antibody-mediated immune response but also compromises the maternal-fetal interface by reducing extracellular matrix proteins and key decidual proteins necessary for the maintenance of early pregnancy.

## Discussion

Successful pregnancy is orchestrated by an interplay of the conceptus, the placenta and the maternal decidua. Decidualization of the endometrium, which occurs during the secretory phase of the menstrual cycle, is crucial for successful embryo implantation and pregnancy [22,23]. Strong evidence indicates that the decidua’s involvement in pregnancy disorders is substantial and impaired decidualization might lead to obstetric complications, such as miscarriage [25].

Studies in reproductive medicine involving humans face practical and ethical hurdles, while animal models often cannot fully mimic unique aspects of human pregnancy, such as spontaneous decidualization and menstruation [22]. Monolayer cell cultures are common *in vitro* models used in biomedical research*,* however, their ability to replicate *in vivo* organ structure and function is limited. Growing cells in 2D results in a loss of tight junctions, abnormal cell polarity and altered gene expression [55,56]. In contrast, 3D cell culture models, such as spheroids, promote cell-cell interactions, resulting in better preservation of the tissue-specific phenotype [50]. We successfully established a novel spheroid model of the human endometrium comprised of stromal and epithelial cell lines using the liquid-overlay technique. In this body of work, we utilized both monolayer cell culture and spheroids to study viral host-pathogen interactions in the endometrium.

SARS-CoV-2 infection is mostly dependent on two key entry factors: ACE2 as the receptor and TMPRSS2 for activation of the spike protein [57]. Our data shows that both these elements are present in endometrial cells and that decidualization increases levels of ACE2 in both stromal and epithelial cells. Likewise, other studies also verify an increase in ACE2 RNA and protein levels after *in vitro* decidualization and *in vivo* [26,58]. It was previously described that ACE2 expression is highest during the secretory phase of the menstrual cycle [59] and during early pregnancy [60], which aligns with our observed increase through decidualization. Notably, loss of ACE2 results in impaired decidualization [26] and reduced ACE2 levels are associated with preeclampsia [61]. Furthermore, SARS-CoV-2 can enter host cells by using other co-receptors [62–64]. According to our reanalysis of single-cell sequencing data, there was no expression of CD209, C-type lectin domain family 4 member G (CLEC4G) and alanyl aminopeptidase (ANPEP) in the endometrium (data not shown). Taken together, ACE2 and TMPRSS2 are the main receptors necessary to facilitate infection in the endometrium.

To assess the susceptibility of the human endometrium to SARS-CoV-2, we infected endometrial monolayers and spheroids. In keeping with other groups, we show that ACE2 and TMPRSS2 increases upon decidualization and thereby permissiveness to SARS-CoV-2. Infection was observed in monolayer ISK cells and endometrial spheroids. Susceptibility to SARS-CoV-2 was highly increased upon decidualization treatment in ISK cells, although comparable amounts of SARS-CoV-2 nucleocapsid protein in both 2D and 3D cultures were seen. This may be due to limitations of the Western blotting technique and due to the different amounts of ACE2 and TMPRSS2 levels in the mixed cell population within the spheroids. Despite this, we revealed that SARS-CoV-2 can infect endometrial cells. Both non-decidualized and decidualized spheroids were susceptible to infection and expressed comparable amounts of SARS-CoV-2 nucleocapsid protein. Thus, our results support the likelihood of SARS-CoV-2 vertical transmission.

The primary aim of the present study was to investigate the potential for SARS-CoV-2 infection within the human endometrium and therefore, the risk of vertical transmission. Several meta-analyses have now been conducted indicating strong evidence of *in utero* transmission of SARS-CoV-2 in around 3% of pregnancies recorded [65]. These studies described a strong correlation of maternal disease severity with SARS-CoV-2 positivity in the fetus/offspring [65]. Furthermore, miscarriage, preterm birth, stillbirths and neonatal deaths were also associated with SARS-CoV-2 positivity [65,66]. Interestingly, a case study revealed that persistent placental infection (placenta was positive for nucleocapsid) was observed in an asymptomatic woman at eight weeks of gestation, leading to fetal demise [15]. It is tempting to speculate that SARS-CoV-2 can remain in non-respiratory tissues (such as the endometrium) [67] and contribute to the post-acute sequelae of SARS-CoV-2, though this conjecture remains to be tested and it remains unknown if this contributes to a novel "category" of unexplained miscarriages. A limitation of the above-mentioned studies is the timing of maternal infection and testing in neonates, maternal ethnicities, co-morbidities, regional access to healthcare and the emergence of new SARS-CoV-2 variants. The low SARS-CoV-2 rates in fetuses/neonates in studies from Europe and North America could reflect the universal maternal screening or shielding from SARS-CoV-2 policies, resulting in inclusion of women with mild disease [68]. Moreover, SARS-CoV-2 RNA has now been confirmed to be present in amniotic fluid, placenta, vaginal fluid and breast milk, but detection of virus in these tissues/fluids may also not necessarily indicate (vertical-)infection of the fetus [65]. Therefore, the inclusion/exclusion criteria as well as the lack of reporting in studies could diminish the observed association between these risk factors and vertical transmission.

Excessive production of proinflammatory cytokines, a phenomenon known as the ‘cytokine storm’, is central to COVID-19 pathophysiology [69]. Hence, we examined the cytokine secretion patterns from endometrial spheroids after infection. Interestingly, we found an increased secretion of MCP-1 by infected endometrial spheroids. MCP-1, a proinflammatory chemokine that promotes macrophage recruitment [70], is found to be higher in COVID-19 patients with intensive care unit (ICU) admission compared to non-ICU patients [71]. Increased levels of MCP-1 in the amniotic fluid during pregnancy are also associated with pregnancy loss and preterm birth with or without infection [72,73]. We further detected increased levels of another chemokine IL-8 after decidualization. This finding is in accordance with a known increase in IL-8 in the mid- and late-secretory phase of the menstrual cycle [74]. Whilst we also assayed other cytokines frequently elevated in SARS-CoV-2 infection, such as IL-6 or tumor necrosis factor (TNF) [75], they were undetectable when using the 3D spheroid model. This may be due to the predominantly immune cell-driven cytokine overproduction observed in COVID-19 [76]. Excessive MCP-1 and IL-8 signaling in the decidua can induce a proinflammatory environment which in turn can impair the local extracellular matrix dynamics at the feto-maternal interface and could compromise pregnancy [77–79]. In keeping with this, the decidua from a SARS-CoV-2 pregnancy also had increased fibrin deposition with extensive leukocyte infiltration suggestive of inflammation and was associated with first trimester demise [15]. Our findings suggest an inflammatory response in endometrial spheroids during SARS-CoV-2 infection could, therefore, result in a cellular state incompatible with the formation of a functional decidual–placental interface which could result in miscarriage.

To analyse the effect of SARS-CoV-2 infection and decidualization on the endometrial spheroids, RNA sequencing was performed. Gene set enrichment analysis identified enrichment in genes associated with immune response, cellular structure and cell metabolism. Several immunomodulatory cytokines and chemokines were dysregulated after viral infection and three of the tested genes were downregulated after infection in non-decidualized spheroids but not in the decidualized spheroids. The chemokine CCL20 is known to regulate immunotolerance as well as inflammation by recruiting immunosuppressive and proinflammatory T cells [80]. CCL20 has direct antimicrobial and antiviral properties [81,82] and the lack of its receptor causes a dampened humoral immune response to rotavirus infection [83]. Contrary to our findings, CCL20 was previously reported to be elevated in the lungs of COVID-19 patients [84]. Similarly, CD38 also plays a vital role in innate and adaptive immunity [85]. Our finding of reduced *CD38* expression upon infection is critical as Henriquez et al. reported a higher risk for SARS-CoV-2 infection despite vaccination in patients undergoing anti-CD38 therapy [86]. LCN2 is likewise a part of the innate immune system with bacteriostatic effects [87]. LCN2 is also involved in the immune response against *Chlamydia trachomatis*, a bacterium known to infect the female reproductive tract [88]. Notably, non-decidualized and decidualized endometrial spheroids were affected differently by SARS-CoV-2 infection. The reason why *CCL20*, *CD38* and *LCN2* were not downregulated in infected decidualized endometrial spheroids might be due to their induction during early pregnancy [89–91]. Furthermore, *LEFTY1* was upregulated in the decidualized spheroids and infection did not significantly impact its expression. *LEFTY1*, is a member of the transforming growth factor (TGF)-β superfamily and is important for left-right patterning during development [92,93]. In support of our finding, *LEFTY1* is known to be upregulated during decidualization [94,95]. In addition, Tang et al., have shown that an increase of *LEFTY* expression was prematurely increased during the implantation window in women with infertility [96].

*VTCN1*, a gene in endometrial spheroids, was downregulated by both decidualization and infection. VTCN1 suppresses T-cell immunity [97] and is associated with immune evasion in cancer [98]. Our observation of its downregulation during SARS-CoV-2 infection aligns with effects seen in *Toxoplasma gondii* infection [99], possibly enhancing the adaptive immune response. We further observed that SARS-CoV-2 infection increases levels of *NR4A3*, which encodes for an intracellular transcription factor [100]. Regarding infection, little is known about the role of *NR4A3*, but a study by Phelan et al. found that its loss might be associated with heightened interferon and viral response [101]. Therefore, upregulation of *NR4A3* might suppress the local endometrial immune response against SARS-CoV-2.

These observations imply that SARS-CoV-2 not only can infect the human endometrium but can also elicit an inflammatory response and alteration of the immune response. During early pregnancy, the endometrium adopts a proinflammatory state and mounts strong innate immune responses, which is vital in combating viral infections [102]. As previously described, SARS-CoV-2 impairs the early interferon response, allowing unchecked viral replication [103]. This can lead to hyperinflammation and the "cytokine storm" seen in severe COVID-19 cases [104]. Our results reveal a potential mechanism of an impaired innate immune response by SARS-CoV-2 in the human endometrium. Intriguingly, this dysregulation appears less prominent in decidualized spheroids.

How the pulmonary virus reaches extrapulmonary sites such as the placenta is an enigma. Ascending vaginal and haematogenous infections are two possible routes. Genital infection, necessary for ascending transmission of pathogens, was not found in SARS-CoV-2 [105,106] and the risk of intrapartum transmission is not elevated by vaginal delivery [107]. Haematogenous transmission requires at least transient viral presence in the blood and viremia has been reported for SARS-CoV-2, albeit at low rates [108,109]. Hence, *in utero* transmission of SARS-CoV-2 likely occurs haematogenously through ‘stochastic seeding’ [67].

The absence of embryonic/placental tissue in our model is a relevant limitation in our study. Future work may involve co-culturing endometrial spheroids with embryonic/placental cells, blood vessels or immune cells for a more accurate maternal-fetal interface representation. Additional research would need to investigate the role of the other SARS-CoV-2 variants to better understand vertical transmission mechanisms and to validate our model.

## Conclusion

The key entry factors of SARS-CoV-2; TMPRSS2 and ACE2, are expressed in the human endometrium and the latter is upregulated during decidualization. Endometrial spheroids can be successfully generated and are a suitable model for studying host-pathogen interactions of the human endometrium. Endometrial cells can be infected with SARS-CoV-2 and infection leads to a dysregulated inflammatory response and a reduced decidual response. Thus, in the eventuality that SARS-CoV-2 is vertically transmitted, it could result in a cellular state incompatible with the formation of a functional decidual–placental interface, potentially leading to miscarriage.

**Clinical perspectives**

- SARS-CoV-2 infection poses a risk to pregnant women and reports of vertical transmission exist. However, data on early pregnancy are limited and the question of in utero SARS-CoV-2 transmission remains unresolved.
- SARS-CoV-2 infects human endometrial monolayers and spheroids. Infection elicits an inflammatory response and dysregulates genes associated with the innate immune system (*CCL20, CD38, LCN2* and *NR4A3*).
- Endometrial inflammation and dysregulated gene expression caused by SARS-CoV-2 infection might play a pivotal role in obstetric complications observed in pregnant women with COVID-19.

## Supplementary material

Supplementary Figure S1

Supplementary Figure S2

Supplementary Figure S3

Supplementary Figure S4

Supplementary Figure S5

Supplementary Figure S6

Supplementary Figure S7

Supplementary Table S1

## Data Availability

Data, code and associated protocols are available upon request to the corresponding author (MSS). Raw data of spheroid RNA-sequencing were deposited into the NCBI database (GEO: GSE274209).

## Abbreviations

ACE2: angiotensin-converting enzyme 2
ANOVA: analysis of variance
DCC: dextran-coated charcoal
DEGs: differentially expressed genes
DGE: differential gene expression
FBS: fetal bovine serum
FDR: false discovery rate
GSEA: gene set enrichment analysis
HESC: human endometrial stromal cells
ICU: intensive care unit
IF: immunofluorescence
IGV: Integrative Genome Viewer
ISK: Ishikawa
MOI: multiplicity of infection
MPA: medroxyprogesterone acetate
NGS: normal goat serum
RNA-seq: RNA sequencing
RT: room temperature
TMPRSS2: transmembrane protease serine 2
WB: Western blot
hpi: hours post-infection
