# Supplementary figures and images for: Harnessing 2D and 3D human endometrial cell culture models to investigate SARS-CoV-2 infection in early pregnancy
Source: Clin Sci (Lond). 2025 Feb 19;139(4):287–307. doi: 10.1042/CS20241215 (PMC12204006; doi:10.1042/CS20241215)

**A**

## Bulk tissue gene expression for ACE2 (ENSG000001340234.10)

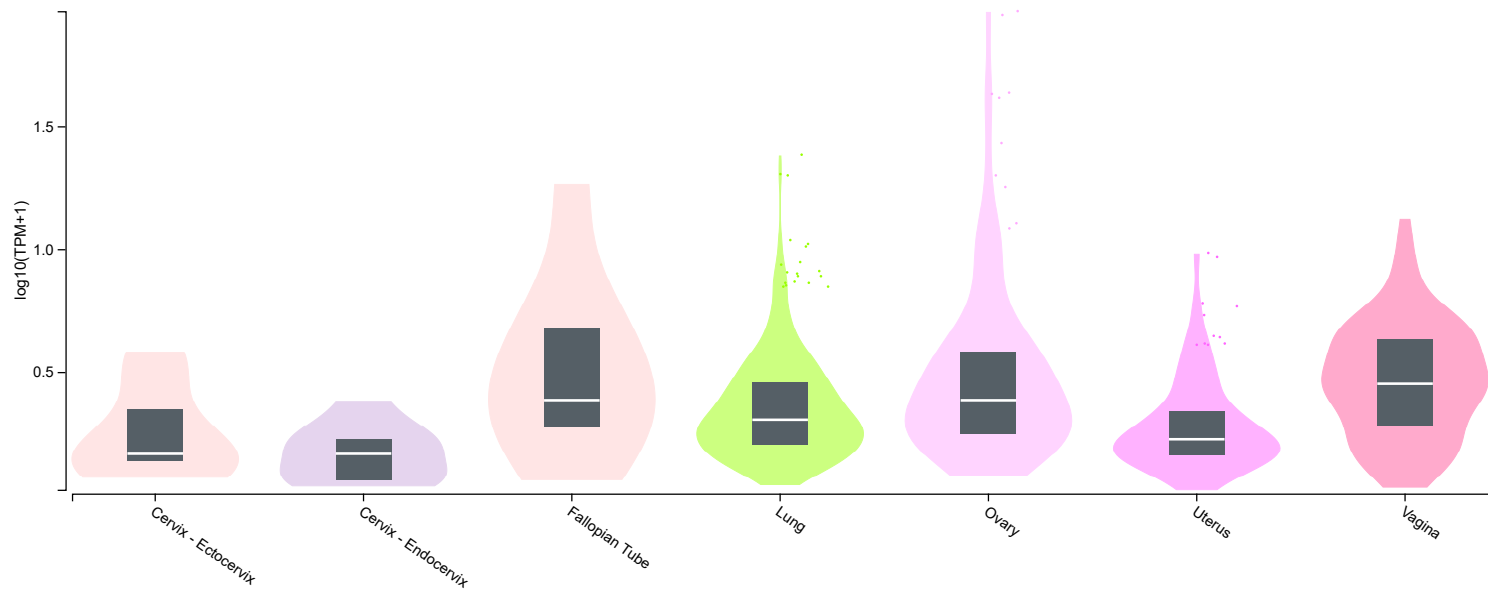**B**

## Bulk tissue gene expression for TMPRSS2 (ENSG00000184012.11)

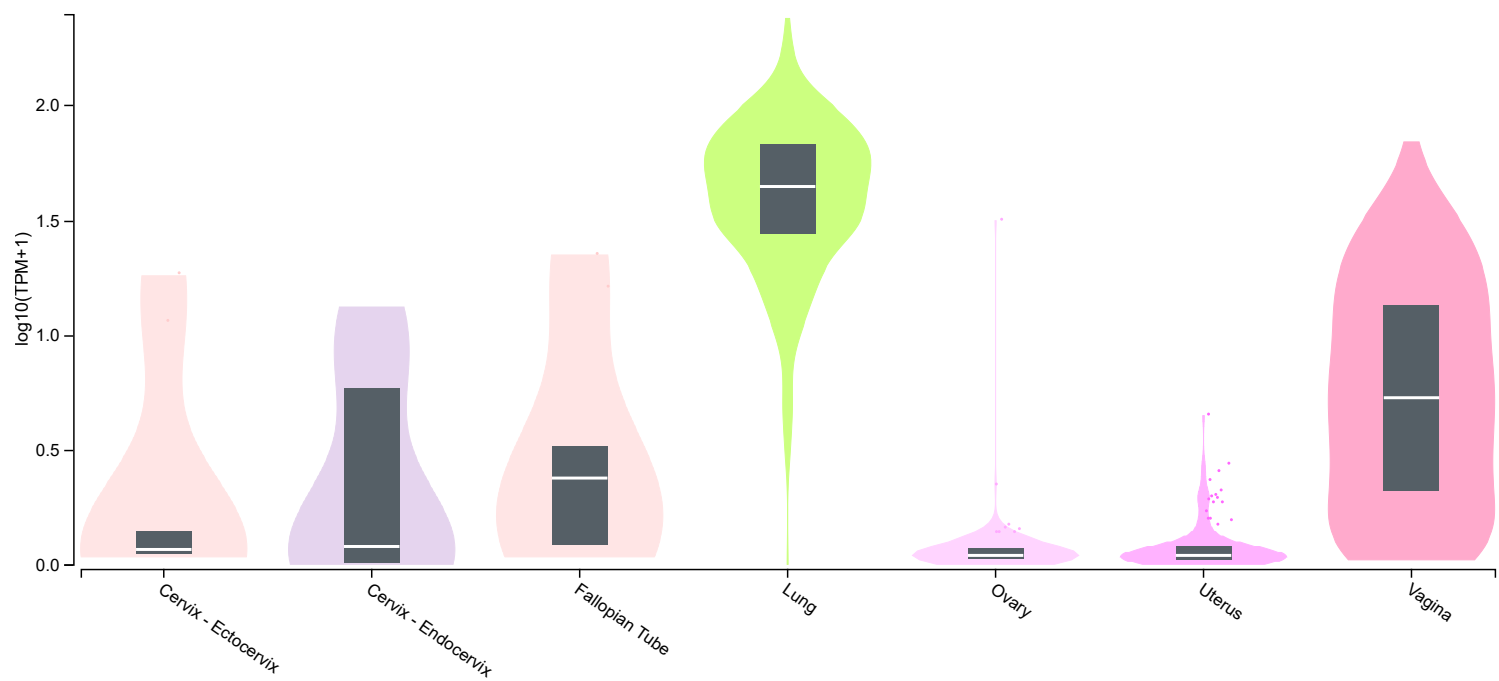

Supplement: Supplementary Figure S1 [file CS-139-04-CS20241215-s001.pdf]

**A**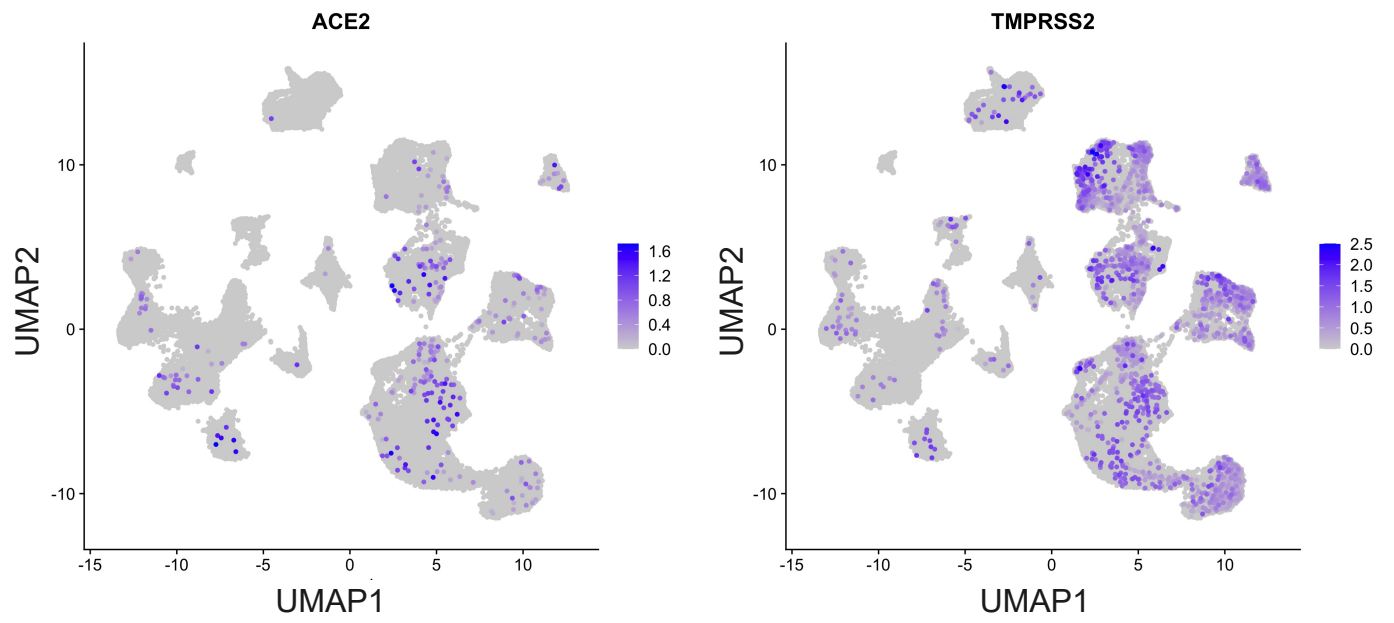**B**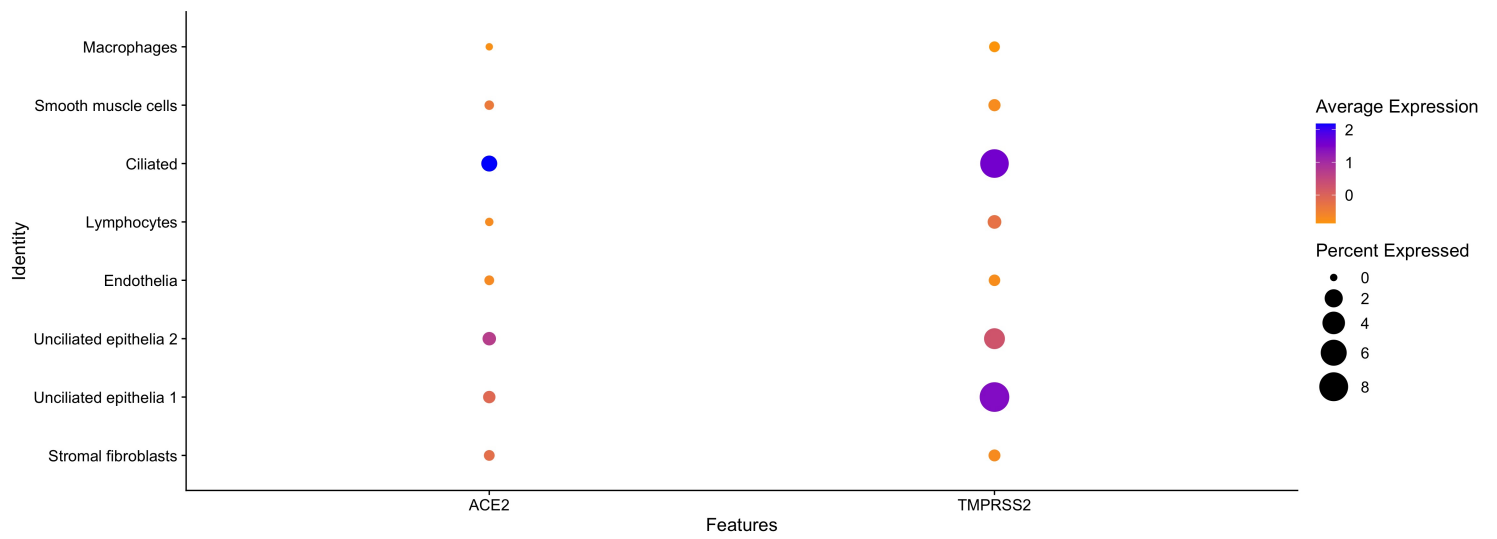

Supplement: Supplementary Figure S2 [file CS-139-04-CS20241215-s002.pdf]

A

HESC

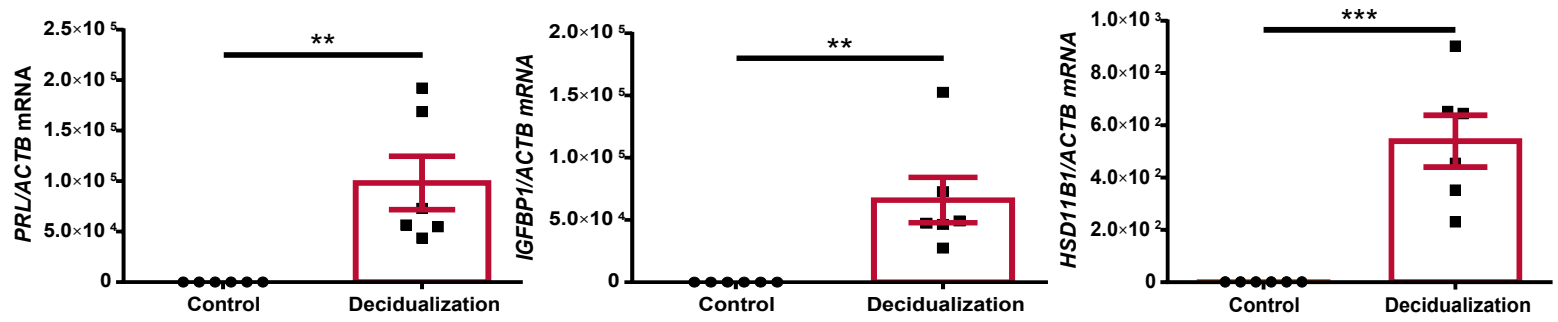

B

ISK

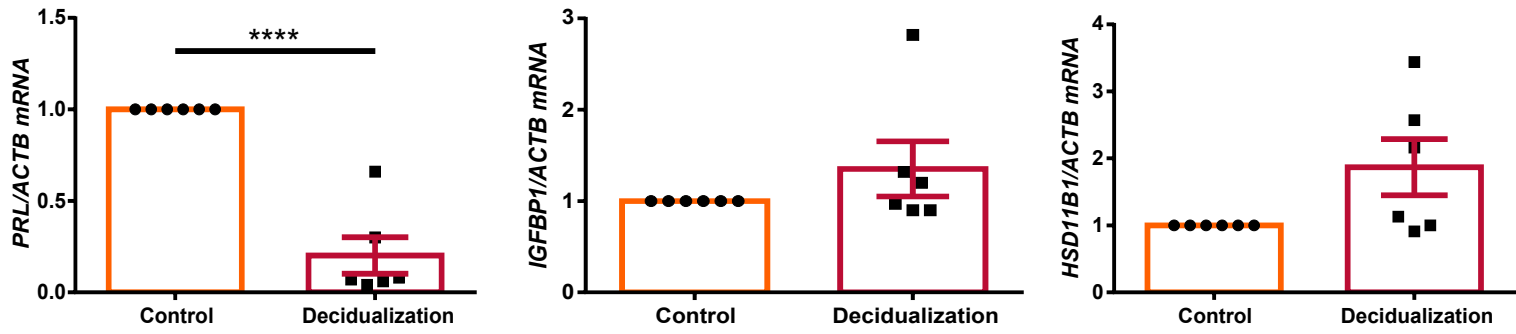

Supplement: Supplementary Figure S3 [file CS-139-04-CS20241215-s003.pdf]

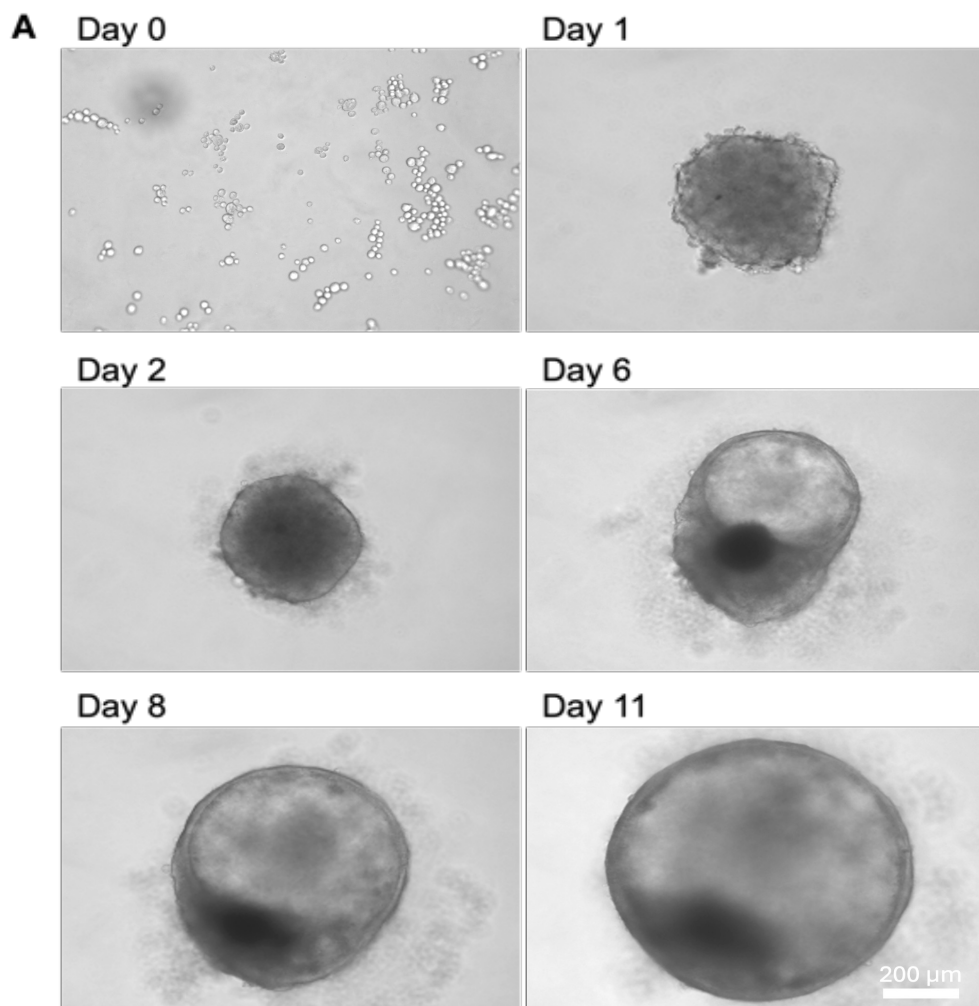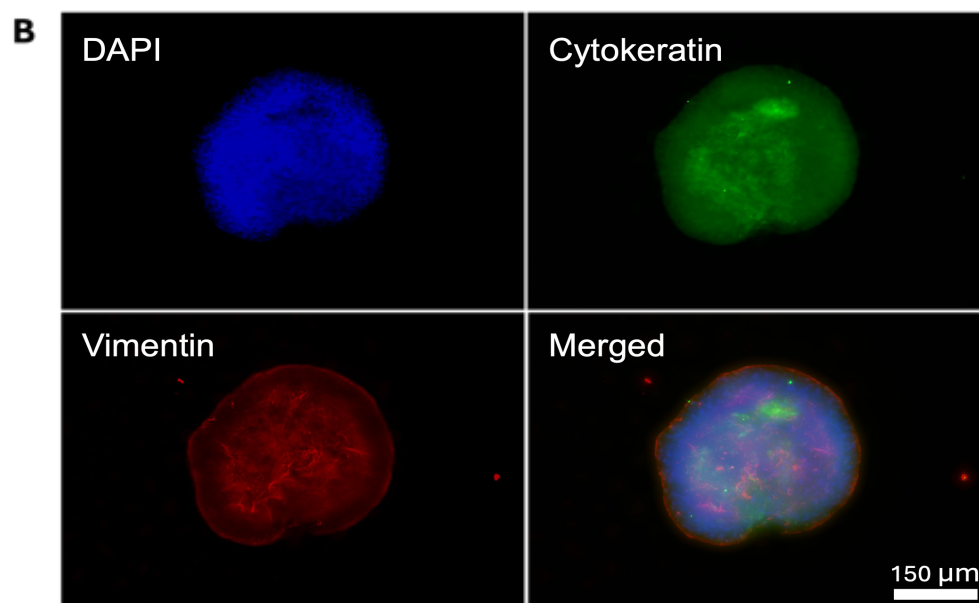

**C**

### Spheroids

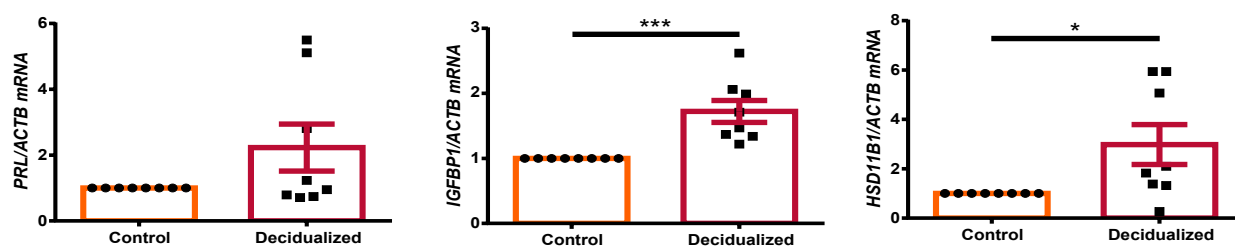

Supplement: Supplementary Figure S4 [file CS-139-04-CS20241215-s004.pdf]

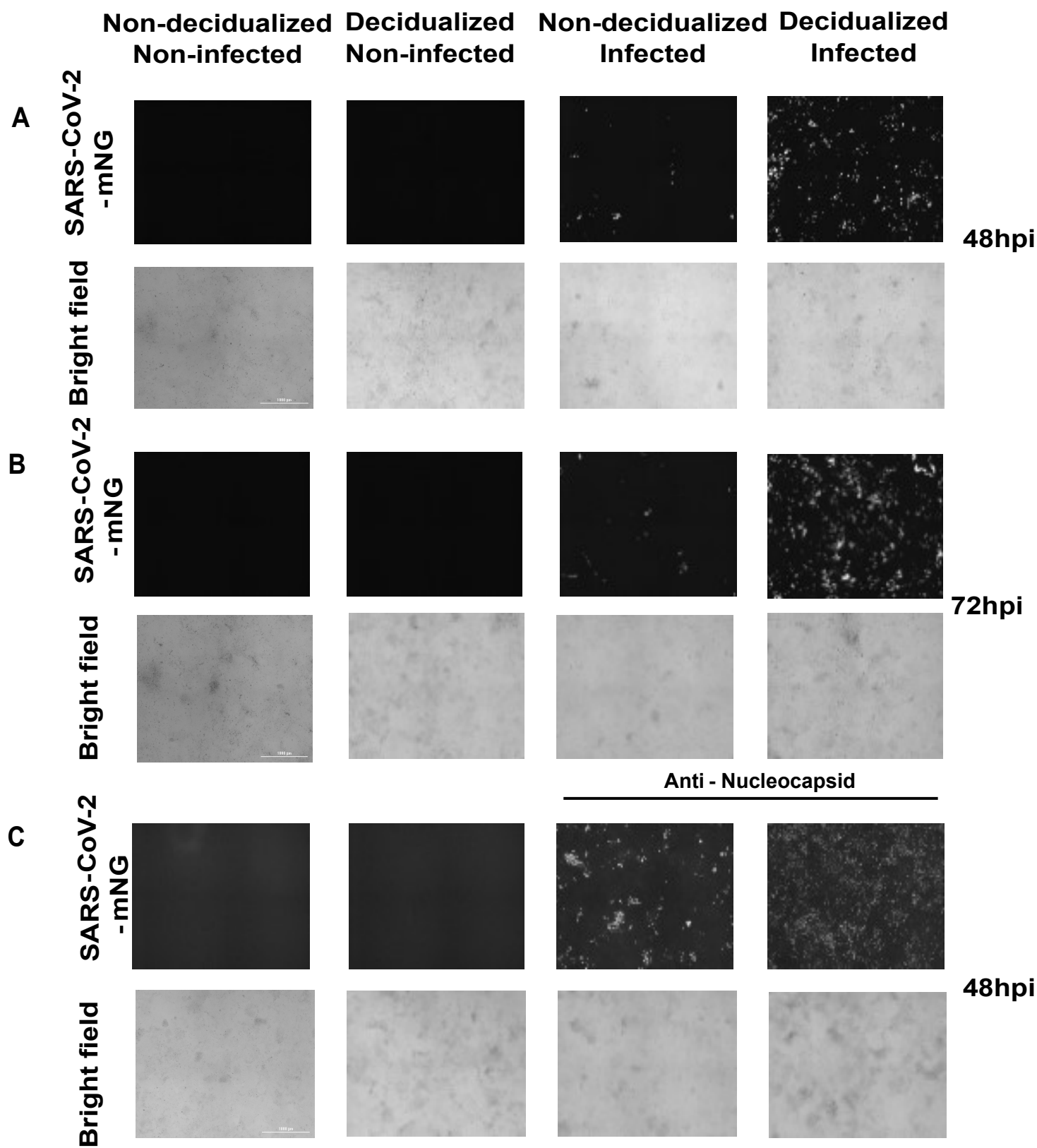

Supplement: Supplementary Figure S5 [file CS-139-04-CS20241215-s005.pdf]

A

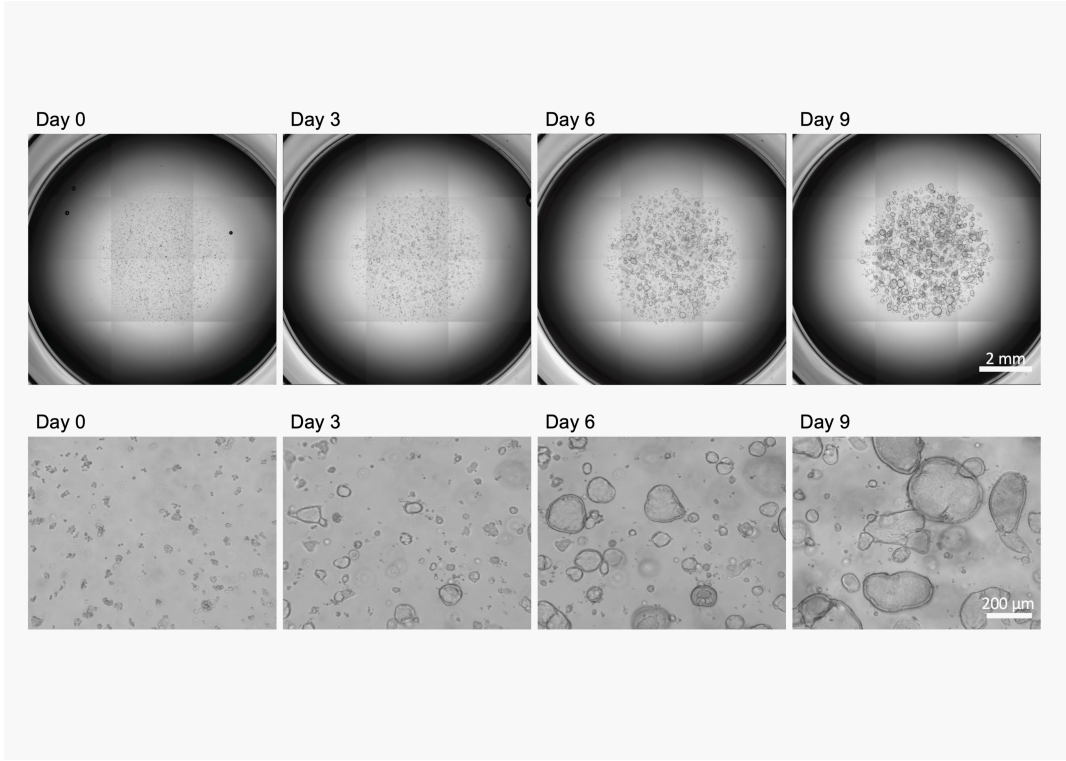

B

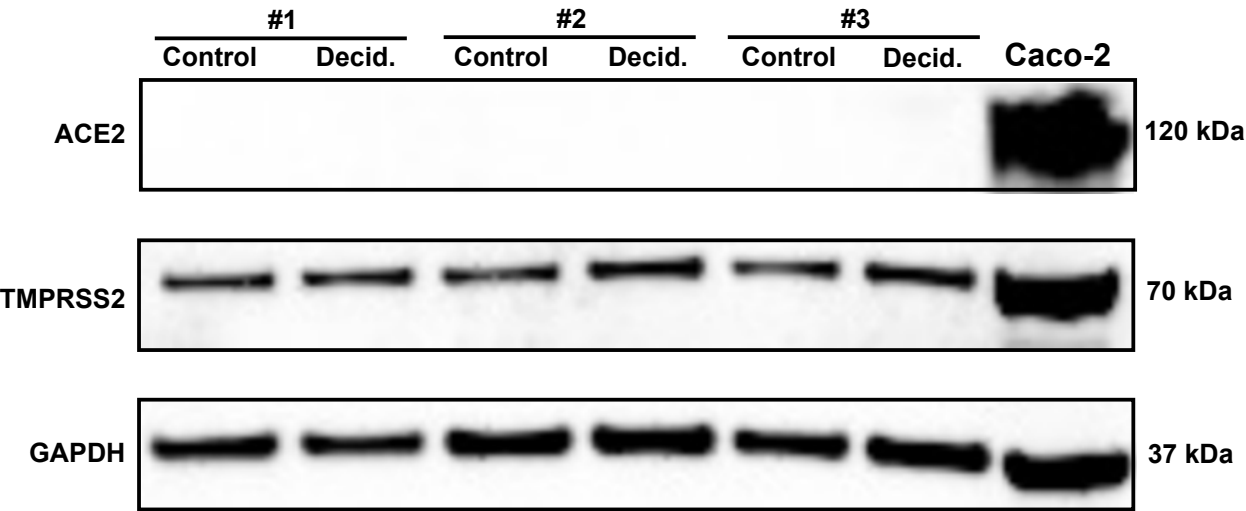

Supplement: Supplementary Figure S6 [file CS-139-04-CS20241215-s006.pdf]
