# Supplementary material for: Harnessing 2D and 3D human endometrial cell culture models to investigate SARS-CoV-2 infection in early pregnancy
Source: Clin Sci (Lond). 2025 Feb 19;139(4):287–307. doi: 10.1042/CS20241215 (PMC12204006; doi:10.1042/CS20241215)
Supplement: Supplementary Figure S7 [file CS-139-04-CS20241215-s007.pdf]

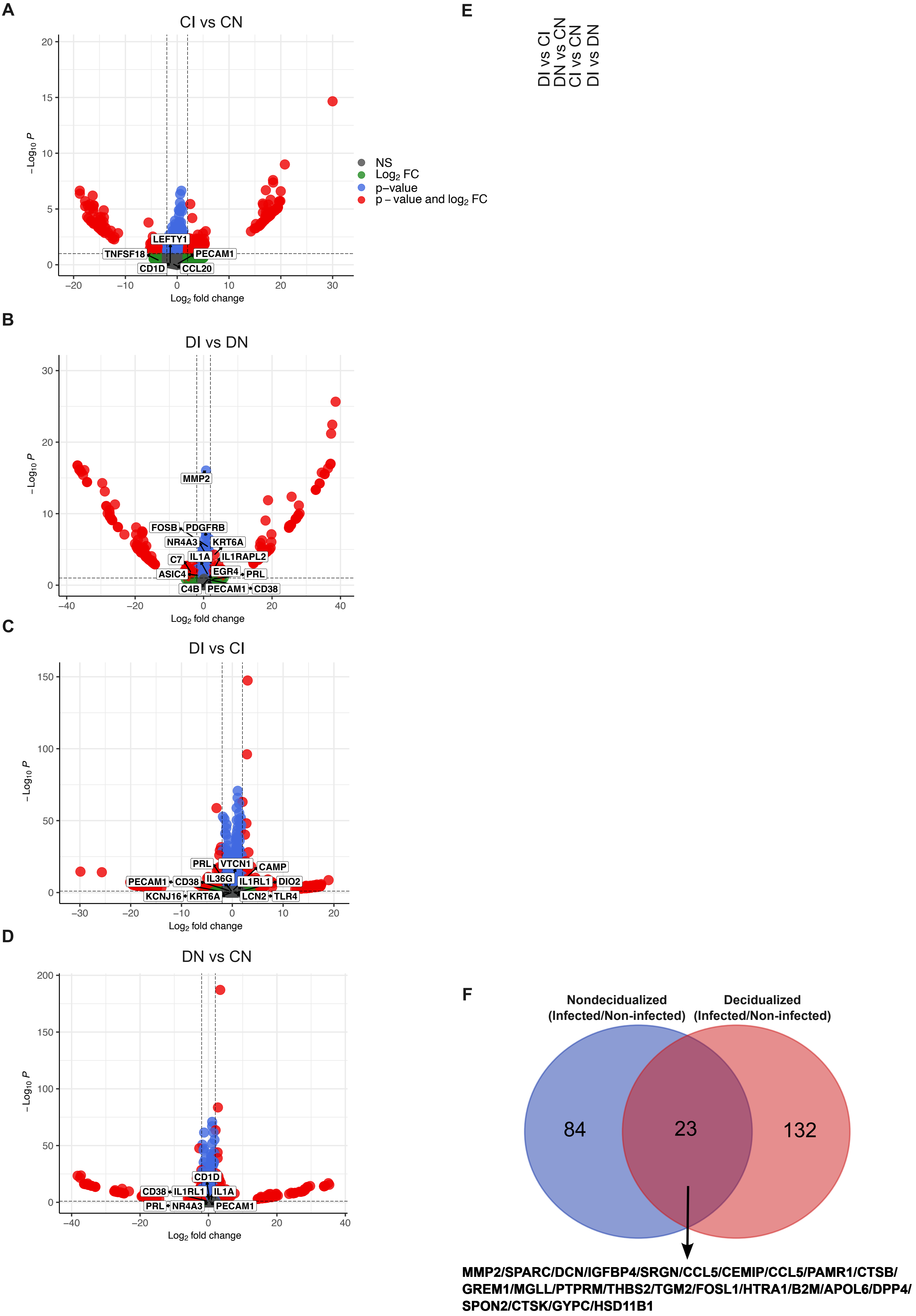
A
CI vs CN
TNFSF18
LEFTY1
PECAM1
CD1D
CCL20
NS
Log2 FC
p–value
p – value and log2 FC
B
DI vs DN
MMP2
FOSB
PDGFRB
NR4A3
KRT6A
C7
IL1A
IL1RAPL2
ASIC4
EGR4
PRL
C4B
PECAM1
CD38
C
DI vs CI
PRL
VTCN1
CAMP
PECAM1
CD38
IL36G
IL1RL1
DIO2
KCNJ16
KRT6A
LCN2
TLR4
D
DN vs CN
CD1D
CD38
IL1RL1
IL1A
PRL
NR4A3
PECAM1
Log2 fold change
–Log10 P
E
DI vs CI
DN vs CN
CI vs CN
DI vs DN
F
Nondecidualized (Infected/Non-infected)
Decidualized (Infected/Non-infected)
84
23
132
MMP2/SPARC/DCN/IGFBP4/SRGN/CCL5/CEMIP/CCL5/PAMR1/CTSB/
GREM1/MGLL/PTPRM/THBS2/TGM2/FOSL1/HTRA1/B2M/APOL6/DPP4/
SPON2/CTSK/GYPC/HSD11B1
